# The development and description of the comparison group in the Look AHEAD trial

**DOI:** 10.1177/1740774511405858

**Published:** 2011-06

**Authors:** The Look AHEAD Research Group

## Abstract

***Background*** Despite more lifestyle intervention trials, there is little published information on the development of the comparison group intervention. This article describes the comparison group intervention, termed Diabetes Support and Education Intervention and its development for the Action for HEAlth in Diabetes (Look AHEAD) trial. Look AHEAD, a randomized, controlled, multicenter trial, was designed to determine whether an Intensive Lifestyle Intervention to reduce weight and increase physical activity reduces cardiovascular morbidity and mortality in overweight volunteers with type 2 diabetes compared to the Diabetes Support and Education Intervention. The Diabetes Support and Education Committee was charged with developing the Diabetes Support and Education Intervention with the primary aim of participant retention.

***Purpose*** The objectives were to design the Diabetes Support and Education Intervention sessions, standardize delivery across the 16 clinics, review quality and protocol adherence and advise on staffing and funding.

**Methods** Following a mandatory session on basic diabetes education, three optional sessions were offered on nutrition, physical activity, and support yearly for 4 years. For each session, guidelines, objectives, activities, and a resource list were created.

***Conclusions*** Participant evaluations were very positive with hands on experiences being the most valuable. Retention so far at years 1 and 4 has been excellent and only slightly lower in the Diabetes Support and Education Intervention arm. The comparison group plays an important role in the success of a clinical trial. Understanding the effort needed to develop and implement the comparison group intervention will facilitate its implementation in future lifestyle intervention trials, particularly multicenter trials. Retention rates may improve by developing the comparison intervention simultaneously with the lifestyle intervention.

## Introduction

There is little information available about the development of the comparison group intervention or its ultimate format and content for large trials of lifestyle interventions despite the recent increase in such trials [1,2]. The scientific value of a randomized trial design and the value of the comparison group are well known and retention of the comparison group is clearly important, as a higher attrition rate in this group jeopardizes the entire study. Investigators are challenged with offering an intervention which produces optimal retention in the comparison group without inducing an intervention effect, and maintaining equipoise [3,4]. For lifestyle intervention trials, the decisions about the comparison group can be even more complex when the trial is multicenter and multi-cultural.

The design of the comparison group intervention for the Look AHEAD (Action for HEAlth and Diabetes) trial, termed the Diabetes Support and Education Intervention (DSEI), was a learning opportunity which has yielded valuable information for investigators. Knowledge and implementation of the strategies used in this trial may benefit investigators in the critical areas of participant retention as well as staff and investigator effort. A brief description of the DSEI in the Look AHEAD study was included as part of the overall design paper [5] and a complete description of the Look AHEAD Intensive Lifestyle Intervention has been published [6]. The objectives of this article are to describe the: (1) development of the Look AHEAD DSEI, (2) content and delivery of DSEI sessions, (3) methods used to standardize the DSEI across sites, and (4) challenges, retention, and lessons learned.

### Description of Look AHEAD

Look AHEAD is a National Institutes of Health (NIH)-funded prospective randomized controlled trial of 5145 people, designed to test the primary hypothesis that an Intensive Lifestyle Intervention to reduce weight and increase physical activity will attenuate the rate of cardiovascular morbidity and mortality in an overweight and obese population with type 2 diabetes [5]. The trial will compare the long-term effects of an Intensive Lifestyle Intervention to that of the DSEI. Within a U01 funding mechanism, the protocol was developed over 16 months by the Look AHEAD Steering Committee and approved by the Look AHEAD Executive Committee, and the National Institutes of Diabetes Digestive and Kidney Diseases. Recruitment began in mid-2001 and was completed in April 2004. Study completion is anticipated in 2014.

## Diabetes Support and Education Committee

**Figure 1:**
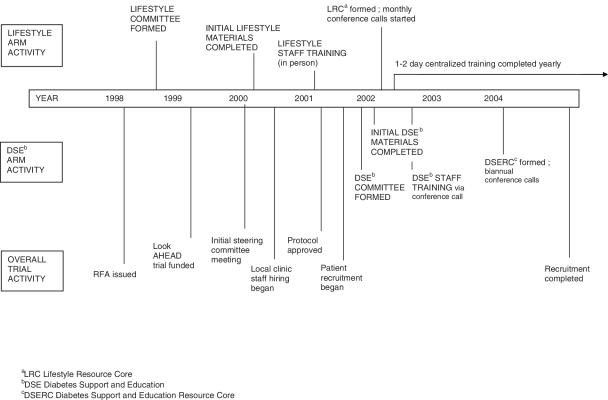
Look AHEAD Trial Timeline

The Diabetes Support and Education (DSE) Committee was formed after the protocol was approved (Figure 1, Timeline). The committee was given the charge of designing a realistic, achievable, and acceptable intervention for the DSE study participants and monitoring clinics to assure standardized delivery. The main goal of the DSE Committee was to maintain a high retention rate for the DSEI participants given the planned follow-up of up to 13.5 years. Thus, the committee specified the following objectives: (1) design and develop interactive DSEI sessions, (2) write and revise the DSEI section of the Manual of Procedures, (3) standardize delivery of the DSEI, (4) review attendance reports and clinic performance, (5) advise the Steering Committee on DSEI matters such as staffing and funding, (6) write the DSEI sections of newsletters, and (7) select and purchase retention items for DSEI participant sessions and annual retention items for all participants.

DSE Committee members included endocrinologists, internists, nurses, dietitians, diabetes educators, exercise physiologists, and behavioral psychologists from several different Look AHEAD sites, who represented the roles of Principal Investigator, Co-Investigator, Project Coordinator, Lifestyle Interventionist, DSEI Facilitator, and DSEI Coordinator. The committee met monthly either by conference call or in-person.

## DSEI session development

### Focus groups

During the planning phase of the study, a public relations firm, contracted by the Look AHEAD study, convened focus groups of individuals who were interviewed as if they were participants randomized to the DSEI. These potential participants were eager to participate in such a trial, and interested in receiving weight loss information and support, but expressed disappointment, anger, and frustration at the concept of being randomized to the DSEI. Based on these results, the DSE Committee was charged with incorporating weight loss information and support into the DSEI curriculum without providing behavioral feedback. This approach was judged to be consistent with providing a beneficial and relevant, yet minimal, intervention unlikely to produce substantial improvements in weight or fitness.

### Curriculum development

The primary goal of the sessions was retention of the DSEI participants by: (1) providing an enjoyable and valuable learning experience; (2) creating a bond with their group leader and other participants; and (3) offering group support. Committee members rotated the lead in developing the curriculum for the sessions based on their areas of expertise: Nutrition, Physical Activity, or Social Support. ‘Guidelines and Teaching Objectives’ and a ‘Resource List’ was created for each session. These two documents listed the course content, session materials, reference materials, patient education materials and resources, and suggestions for retention items.

Since Look AHEAD clinics are located across the USA, and included 2575 individuals randomized to the DSEI, the DSE Committee wanted to ensure that materials were written at an appropriate literacy level and were culturally sensitive. Thus, the DSE Committee reviewed all the DSEI materials for content, adherence to a 7th grade literacy level[7], and cultural sensitivity. A ‘Literacy Tip Sheet’ was developed to assist committee membersin developing materials at the appropriate reading level using the SMOG Tool [7] for assessing readability. Several members of the DSE Committee attended cultural diversity training at the beginning of the study. By utilizing the knowledge gained by these members’ and the diversity of the committee members, an effort was made to consider attitudes, behaviors, and beliefs important to the various minorities represented in the study. For example, Native American participants who were shepherds and walked all day needed a different approach to understanding the need for physical activity compared to urban dwelling participants. Recipes and cooking demonstrations were flexible to allow inclusion of cultural food preferences or alternative preparation styles. Materials were made available to all clinics via an intranet website. The usual timeframe for developing and finalizing teaching materials for a session was 4–6 months.

### DSEI staffing

Each Look AHEAD clinic identified one DSEI Coordinator and 1–3 DSEI Group Leaders for their site. DSEI Coordinators and Group Leaders included nurses, dietitians, exercise specialists, and diabetes educators. Each clinic was permitted to select the most qualified professionals to facilitate the sessions. The DSEI Coordinator oversaw certification of staff and conduct of the DSEI and was responsible for DSEI participant tracking and retention. Since Look AHEAD participants were randomized in groups or cohorts, a DSEI Group Leader was assigned to each group and expected to attend all of that group’s sessions and serve as the contact person for participants to enhance bonding and, ultimately, participant retention. Both DSEI Group Leaders and DSEI Coordinators could serve as Facilitators of DSEI sessions at different times.

### Session delivery and content

The DSEI was designed to be delivered in small group settings of up to 20 participants. Around the time of randomization, all subjects received a session on the key aspects of diabetes self-care and safety. Subsequently, the DSEI participants were offered three sessions, 60–90 min in length, annually for the first 4 years of follow-up; thereafter, one session was provided annually.

In year 1, the Nutrition and Physical Activity sessions were delivered in a didactic classroom setting and provided basic information on nutrition and physical activity essential to all people with type 2 diabetes. The year 1 Social Support session provided a forum for participants to discuss and share feelings, concerns, attitudes, and beliefs about living with diabetes as well as their randomization to the DSEI. Subsequent sessions in years 2–4 had a central topic, but clinics were provided a menu of educational activities from which DSEI Facilitators could select. They were designed to be interactive (Table 1). Each clinic could adapt the session to their clinic setting and the culture of their participants, but individualized behavioral feedback and follow-up were *not* permitted. When participants raised questions about their individual care, Coordinators and Group leaders were trained to respond in general terms and/or encourage participants to follow-up with their own doctor or other care provider. (Clinical and laboratory data from annual study visits were sent to each participant’s doctor.) Handouts of the information covered were provided at each session.

**Table 1:** DSEI^a^ sessions, topics and retention items

Year	Session	Topics	DSEI^a^ retention items
1	Nutrition	Basic nutrition for type 2 diabetes	Measuring cups^b^
	Physical activity	Basic physical activity for type 2 diabetes	Exercise bands, sport socks^b^
	Social support	Open support session on difficulties in living with diabetes	Stress balls^b^
2	Nutrition	Low fat cooking, high fat recipe modification, and cooking with spices or label reading	Insulated lunch bags^b^
	Physical activity	Foot care or exercise session with exercise bands	Sport towels^b^ and foot inspection mirrors^b^
	Social support	Stress management	Relaxation book and tape^b^
3	Nutrition	Eating out; our changing environment or supermarket tour	Egg separator^b^ and pot holder^b^
	Physical activity	Exercise and diet fads or working out at home	Low-impact exercise video and book
	Social support	Stress and eating	Spiral notebook^b^ and ink pen^b^
4	Nutrition	Low-carbohydrate diets, popular diets, or glycemic index	Coffee mug^b^
	Physical activity	Exercise sampler or fitness facility tour	Fanny pack^b^
	Social support	Motivation and changing behavior	Gentle timer reminder and bedside illuminated notepad

a DSEI – Diabetes Support and Education Intervention.

b Look AHEAD logo placed on item.

Sessions were organized by session year (Table 1), but clinics were permitted to deliver the sessions in any order for any given year. Each clinic was required to record participant attendance on the study site intranet. The clinic staff and the DSE Committee then were able to review attendance by participant identification number and clinic. Participants were permitted to make up (in the year of study participation) any sessions they missed. On average, participants attended 2.9 sessions in year 1 (including the required safety session), 1.6 in year 2, 1.4 in year 3, and 1.1 in year 4.

### Participant contacts

Under the direction of the DSE Committee, the public relations firm created fliers to announce thedate, time, and content of each session which were mailed to DSEI participants. Clinic staff were encouraged to make reminder calls 1 week prior to each session. After a session, clinic staff contacted participants who did not attend to reschedule and/or mail the materials to participants. Thus, counting all of the study-related communication, each DSEI participant received a minimum of 13 contacts per year (Table 2). Participants were reminded regularly of how important their role was in determining whether the required long-term lifestyle changes were really beneficial and how they were making a contribution to benefit all of society.

**Table 2:** Contacts with Look AHEAD participants assigned to the comparison group

*Required*
Three informational and support sessions offered per year of participation
(one repeat if participant desires)
Maximum of two social events
One semi-annual phone contact for data collection
One annual clinic visit
One birthday card
One holiday card
One annual calendar
Four newsletters per year (one National and three Local)
*Optional*
Flyers mailed before each session
Session reminders-by mail or phone
Social phone calls (i.e., death in the family, loss of employment, birth of a grandchild, etc.)
Other contacts (i.e., greeting cards and mailing of retention gifts, etc.)

### Retention items

The DSE Committee identified items intended to encourage retention (Table 1) to be given to participants in each session. These were chosen to fit in with the session content and have broad appeal and usefulness. Each clinic was encouraged to send these items to participants who did not attend. The Coordinating Center purchased all the incentives (generally less than $5 per item) to take advantage of large discounts, and shipped them to each clinic. Suggestions for additional incentives were provided; however, the choice and purchase were left to individual sites to allow for regional and cultural adaptation.

### Session evaluations

At the end of each session, DSEI participants were asked to rate the session and identify topics for future sessions. DSEI Coordinators summarized these comments which were reviewed by DSE Committee members on a regular basis. The feedback was used to evaluate adherence to the protocol, review the global experience of DSEI participants, develop future sessions and identify problematic issues at the clinic level. Suggestions, especially when seen on multiple evaluations, were incorporated into future sessions whenever consistent with the protocol. Issues thought to be site specific were discussed with the responsible DSEI Coordinator at that site. Overall, the evaluations were extremely positive, with the vast majority of ratings being ‘very good’ and ‘excellent.’ In particular, participants stated that ‘hands-on’ experiences such as cooking demonstrations and demonstrations of fit balls, resistance bands and chair exercises were the most valuable.

## Standardization of the DSEI

Since the Look AHEAD DSEI was designed to be delivered by 16 different centers across the USA, the DSE Committee took several steps to standardize content delivery and to establish internal validity. First, use of the Look AHEAD intranet site provided easy access to all materials by DSEI Coordinators and Facilitators and allowed them to be updated quickly. Any changes or new session postings were announced in the monthly Coordinating Center newsletter and emailed to all study personnel.

Second, the DSE Committee developed several documents to provide direction to Facilitators. ‘Leading Diabetes Support and Education Sessions: Background and Tips,’ addressed issues such as group size, length and frequency of sessions, inclusion of significant others in sessions, repeating sessions and use of clinic funds for local retention items. The document, ‘Leading Effective Groups,’ which was available to Intensive Lifestyle Intervention staff, was made available to the DSE staff and addressed the fundamentals of leading groups. Additional session support documents are listed in Table 3.

**Table 3:** Documents provided to DSEI^a^ Facilitators

DSEI^a^ documents	Content of document
Welcome to DSE^b^	Welcome handout for participants reviewing components of DSEI^a^ program
Leading DSE sessions: background and tips^b^	General rules for leading all DSEI^a^ groups
Leading effective groups^b^	Review of fundamentals of leading groups
Guidelines for Weight Comparison Education^b^	Specific information on appropriate weight comparison information to provide to the DSEI^a^
Guidelines for Collapsing Cohorts^b^	Advantages/disadvantages, and procedure for data entry when collapsing groups
Guidelines and Teaching Objectives (for each session)	Facilitator guide and content for their respective session
DSE participant contacts^b^	Detail of number of required and optional contacts
DSE tips	List of successful approaches to DSEI^a^ participants
Literacy Tip Sheet	Summary of guidelines to achieve recommended literacy level for any participant materials produced
Talking Points	Summary of the benefits of the DSEI^a^ program

a DSEI – Diabetes Support and Education Intervention.

b Required for certification.

### Certification

A mandatory certification process for DSEI Facilitators, Group Leaders, and Coordinators was developed to foster consistent delivery of session content and ensure DSEI Facilitators had thorough knowledge of the goals and purpose of the DSEI. The certification process consisted of reading the documents noted in Table 3 in addition to the materials specific to each session. Completion of certification could take 2–4 hours and once completed, was entered on the website. Each time a new session was posted, all DSEI Facilitators were required to complete certification for that session. The DSE Committee and Coordinating Center staffmonitored the certification status of all Facilitators.

### DSE resource core groups

As the clinics began to deliver the DSEI sessions, it was apparent that more training and support for the DSEI Facilitators and Coordinators would improve communication across the sites and address questions and problems in a more proactive manner. A survey of 17 questions was sent to each clinic to clarify exactly how each clinic organized their DSE team and delivered the DSEI. The survey of DSEI Coordinators revealed two important points: (1) many clinics did not appoint a DSEI leader to each DSEI group and (2) many clinics did not have a procedure for follow-up of missed sessions.

Following the survey, a training conference callwas held for groups of 4–5 clinics. The call was mandatory for all DSEI Coordinators and followed an agenda set by the DSE Committee. Results of the survey were discussed and DSEI Coordinators brainstormed on how to implement changes. Subsequently, the DSE Committee established four DSE Resource Core (DSERC) groups, modeled after the previously established Look AHEAD Lifestyle Resource Core groups. The DSERC groups, led by 1–2 DSE Committee members, were designed to enhance standardization, address questions proactively, review problems at the sites and provide a forum for the Coordinators to share experiences. Once initiated, DSERC conference calls led by one of the DSE Committee members were held every 6 months. These conference calls were discussed ahead of time by the DSE Committee and attended by the Coordinating Center representative on the committee. If important information emerged from any group call,theinformation was shared with all DSERC groups.

## Challenges and lessons learned

### Disappointment and anger

One of the main challenges for clinics was the disappointment and anger that some participants expressed when randomized to the DSEI. This reaction is common to lifestyle intervention trials where participants are attracted by the goals of the intervention but are unblinded to their assignment, in contrast to placebo controlled drug trials. Even as time passed, a few participants occasionally expressed disappointment and anger at not being randomized to the Intensive Lifestyle Intervention, despite a group information session and multiple discussions about the meaning of consent to accept random assignment. DSEI Facilitators needed mentoring and support to address these issues with participants. Therefore, the DSE Committee developed several documents to assist the clinic staff (Table 3). For example, one document, ‘Talking Points,’ recommended validating participant feelings, reviewing the purpose of the study, and reminding DSEI participants that they could attempt weight loss on their own.

Designers of future lifestyle intervention trials should consider that, given their unblinded nature, some participants may be more disappointed than anticipated, as the information gathered from pre-study focus groups revealed. Furthermore, these feelings may linger several years into the study. Consideration of preventive actions before or at the time of randomization, and earlier development of guidelines could assist staff to deal with this potential problem. Both of these actions could prevent or limit some of the persistent disappointment among participants which could negatively affect retention.

### Collapsing cohorts

Originally, all participants were divided into groups or cohorts, similar to the Intensive Lifestyle Intervention participants. Not surprisingly, over time, decreasing attendance at the sessions sometimes resulted in too few participants for quality group interaction, a number estimated to be 10–20 for weight loss groups [8,9]. The DSE Committee developed general guidelines for leading and collapsing groups (Table 3). When attendance was low, DSEI Facilitators were encouraged to ‘collapse’ two or more groups into one larger group, provided the sessions did not include more than 20 people. Collapsing cohorts also reduced the staff burden and made for more efficient use of staff time, which was reduced gradually over time. Combining groups allowed participants to attend sessions at different times and on different days. In future trials, when interventions are delivered in group settings, studies should consider proactively collapsing groups as class sizes decrease, in order to maintain quality interaction, enhance retention, and increase staffing efficiency.

### Timely session development

The Look AHEAD Trial Timeline is shown in Figure 1 and details the difference in study activities by study arm. The development of the DSEI began about the time the protocol was completed when recruitment was starting. In contrast, the development of the initial Intensive Lifestyle Intervention sessions was completed before study recruitment began. Thus, the amount of time available to complete the year 1 DSEI sessions (including the processes for literacy and cultural review, guidelines and procedures for dissemination of materials, certification of Facilitators, evaluation of sessions, and attendance tracking) was far less than the time used to develop the Intensive Lifestyle Intervention sessions. This proved to be a difficult task for the DSE Committee. Once the infrastructure and processes were in place, and with a better understanding of the time needed, the DSE Committee started developing sessions earlier, resulting in more timely availability to clinic staff.

While a great deal of time and effort is needed to develop lifestyle interventions, future trials should also begin the design of the comparison group intervention early, ideally in the planning phases of the study. In long-term studies, perhaps greater attention is needed for the comparison group intervention for retention purposes, especially weight loss studies, which typically have had low retention rates [10–12]. An early start allows for greater opportunity to design a program with a ‘perceived benefit.’ Without any valuable feature to the comparison group intervention, lifestyle intervention trials risk the loss of comparison group participants, which can jeopardize the validity of the study. Finally, a clear understanding of the comparison group intervention, including the time and resources needed for development and implementation, is needed among the entire study group. The appropriate oversight committees of multi-center trials need to assure sufficient staffing and resources both centrally and at the local clinics to develop and deliver the comparison group intervention.

### Maintaining communication

Communication with the administrative structure of a large trial is extremely important. Formation of the DSERC groups, albeit belated compared to the Lifestyle Resource Core groups (Figure 1), enhanced communication with and support for the DSEI Coordinators, Leaders, and Facilitators.

Channels of communication among national Look AHEAD Study Committees also were needed. The Lifestyle Intervention Committee often was concerned about providing the DSEI participants too much information, and potentially reducing the difference in intervention variables and outcomes. The DSE Committee was concerned with giving participants a valuable and positive learning experience, while the Retention Committee was concerned with the long-term retention of all participants over the 13.5 years of the study. Finally, the Project Coordinator Committee was concerned with proper staffing of the clinics within budgetary constraints. Communication via conference calls, e-mails, phone calls, and discussions at Executive Committee and Steering Committee meetings was critical in addressing these issues. Ultimately, a Cross-Study Retention Committee was formed, which included members from each of these committees.

Early development of formats for communication among committees, clinics and the study administration can proactively address consistency with study goals and improve staff training, support, and morale. Additionally, these structures can assist clinics with the staff orientation needed due to the inevitable staff turnover that occurs during long-term studies [13].

### Retention of participants

The main aim of the DSEI was participant retention throughout the 13.5 years of planned follow-up. We planned for a more intensive comparison group intervention than many prior studies [14,15] because many weight loss trials have less than 1 year of follow-up (typically 6 months). Even placebo controlled weight loss trials (in which participants are blinded to their treatment assignment) have had retention rates below 90% at 1 year [16] and many achieve less than 70% [10–12]. The DSE Committee worked under the premise that if DSEI participants had a ‘perceived benefit’ from these sessions and formed a closer bond with the study staff, their commitment would be strengthened and retention in annual outcome assessments would be enhanced. However, from the study perspective, a key aim was to produce a difference in weight and fitness between the participants in the two study arms; a goal which was achieved after 1 year (mean weight loss in the Intensive Lifestyle Intervention arm 8.6% versus 0.7% in the DSEI arm, mean increase in fitness 20.9% in the Intensive Lifestyle Intervention arm versus 5.8% in the DSEI arm) [17].

In terms of retention, early numbers suggest success. The 1-year exam was attended by 96.4% of participants, which was only slightly, but statistically significant between the two study arms (Intensive Lifestyle Intervention 97.1% versus DSEI 95.7%; *p* = 0.004) [17]. The 4-year exam was attended by 93.6% of participants. There was no significant difference between the two study arms (Intensive Lifestyle Intervention 94.1% versus DSEI 93.0%; *p* = 0.11) [18]. Such high 1-year and 4-year retention rates in a weight loss trial are remarkable. Furthermore, there was a significant stepwise trend between attending a greater number of DSEI sessions and higher retention at the 1-year visit, with only 85% of those participants who attended no DSEI sessions the first year completing data collection, compared to 99% of those attending all three DSEI classes (*p* < 0.001). While this does not prove causality, it provides face validity that the DSEI sessions were valuable in enhancing retention in the comparison group.

The years 1 and 4 retention data in Look AHEAD equal or exceed those of other large multi-center lifestyle intervention trials (Table 4). The Weight Loss Maintenance trial retained 94.7% of its Self-Directed control group after 30 months; however, this study randomized only patients who had lost 4 kg during a 6-month intervention period [15]. The Diabetes Prevention Program retained 92.4% of its participants at the end of the study (2.8 years); however, the control group in that study was a placebo control group designed in comparison to the metformin and troglitazone arms of the study [19]. Perhaps most similarly, the Finnish Diabetes Prevention Study had an overall retention rate of 97.1% at 1 year, but only 90.1% at 2 years [20]; furthermore, the retention rates by study arm were not reported clearly. Prior to Look AHEAD, the longest lifestyle intervention trial was the Women’s Health Initiative which reported an overall retention rate of 90.8% (90.4% for lifestyle and 91.1% for the comparison group) at 8 years of follow-up [21]. Look AHEAD is currently in year 8 of data collection, so comparison at that time point will be available in the future.

**Table 4:** Comparison of retention rates across large lifestyle intervention trials

Study	Year 1 retention-comparison group	Year 1 retention-intervention group	Year 1 retention-overall	End of study retention-comparison group	End of study retention-intervention group	End of study retention-overall	Trial features
Weight Loss Maintenance Trial	94.7%	95.8%	95.4%	93.6%^a^	93.3%	93.4%^a^	Rates in this table combine the two intervention groups vs the comparison group. The trial only randomized those that lost 4 kg in phase I
Diabetes Prevention Program	Not reported	Not reported	Not reported	Not reported	Not reported	92.4%	Three blinded medication arms, 1 lifestyle arm. Comparison here is placebo group vs lifestyle. Mean followup 2.8 years
Finnish Diabetes Prevention Study	Not reported	Not reported	97.1%	93.4%	91.3%	92.3%	Mean followup was 3.2 years
Women’s Health Initiative (low-fat diet trial)	Not reported	Not reported	Not reported	91.1%	90.4%	90.8%	Intervention involved 18 sessions in first year, then quarterly groups. Subjects may have been in additional subcohorts within WHI^b^. Mean followup was 8.1 years
Look AHEAD trial	95.7%	97.1%	96.4%	93.0%^c^	94.1%^c^	93.6%^c^	

a End of study was 30 months.

b Womens’s Health Initiative.

c Trial is still ongoing; 4-year data were the most recent released.

Tracking both delivery of the DSEI and individual participant attendance was very useful for individual clinics as well as the DSE Committee. Furthermore, making a schedule of planned and optional contacts for the DSEI (Table 2), similar to what was done in the Intensive Lifestyle Intervention, allowed the clinics to monitor the retention rate and be proactive at their sites. The high retention rate so far suggests that the DSEI was successful. Future studies should consider what intensity of efforts will be needed to retain participants in the study, but minimize the effects of such efforts on study outcomes. The potential effects of these efforts on study power should also be considered during the planning phases.

## Conclusions

The DSEI of the Look AHEAD trial required a substantial investment of time and effort from a multi-disciplinary committee in order to develop it in a timely manner, assure appropriate training of staff, monitor delivery at the clinics, address the issues of participant disappointment, and finally, enhance attendance and ultimately retention. Without a ‘perceived benefit,’ studies risk the loss of comparison group participants, particularly in long-term studies, that may jeopardize the entire study. During the first 4 years of follow-up, retention of DSEI participants was very high, but notquite as high as for the Intensive Lifestyle Intervention group. It is not known whether a greater investment in the DSEI early on would have produced equal retention rates for the Intensive Lifestyle Intervention and DSEI groups and the effect on longer-term retention rates remains to be seen. In retrospect, the amount of time and effort needed to develop and implement the Look AHEAD DSEI was underestimated initially. Furthermore, communication strategies across multiple clinic sites and committees were needed to insure consistent efforts toward the overall study goals. Other clinical trials may replicate this process by developing the comparison intervention sessions at the same time as the lifestyle intervention sessions, monitoring delivery, obtaining participant feedback, and providing support for the staff. If these steps are taken earlier, the comparison group retention rate could be even higher than in Look AHEAD.
